# Associations between methamphetamine use disorder and *SLC18A1*, *SLC18A2*, *BDNF*, and *FAAH* gene sequence variants and expression levels

**DOI:** 10.1080/19585969.2024.2413476

**Published:** 2024-10-12

**Authors:** Alexandre A. Guerin, Briana Spolding, Kiymet Bozaoglu, Courtney Swinton, Zoe Liu, Bruna Panizzutti Parry, Trang Truong, Brian Dean, Andrew J. Lawrence, Yvonne Bonomo, Eric J. Nestler, Peter J. Hamilton, Michael Berk, Susan Rossell, Ken Walder, Jee Hyun Kim

**Affiliations:** a Centre for Youth Mental, University of Melbourne, Melbourne, Australia; b Orygen, Melbourne, Australia; c IMPACT, The Institute for Mental and Physical Health and Clinical Translation, School of Medicine, Barwon Health, Deakin University, Geelong, Australia; d Murdoch Children’s Research Institute, Parkville, Australia; e Department of Paediatrics, University of Melbourne, Parkville, Australia; f The Florey Institute of Neuroscience and Mental Health, Parkville, Australia; g Florey Department of Neuroscience and Mental Health, University of Melbourne, Parkville, Australia; h Department of Addiction Medicine, St Vincent’s Hospital, Melbourne, Australia; i Department of Medicine, University of Melbourne, Melbourne, Australia; j Women’s Alcohol and Drug Service, Royal Women’s Hospital, Melbourne, Australia; k Nash Family Department of Neuroscience, Friedman Brain Institute, Icahn School of Medicine at Mount Sinai, New York, NY, USA; l Department of Anatomy and Neurobiology, Virginia Commonwealth University School of Medicine, Richmond, VA, USA; m Centre for Mental Health, Swinburne University of Technology, Melbourne, Australia; n Department of Psychiatry, St Vincent’s Hospital, Melbourne, Australia

**Keywords:** Substance use disorder, single nucleotide polymorphism, VMAT, inhibitory control, cognitive flexibility, endocannabinoid

## Abstract

**Introduction:**

Assessing candidate gene sequence variations and expression helps to understand methamphetamine use disorder and inform potential treatments. We investigated single nucleotide polymorphisms (SNPs) and gene expression in four candidate genes: *SLC18A1, SLC18A2, BDNF,* and *FAAH,* between controls and people with methamphetamine use disorder.

**Methods:**

Fifty-nine participants (29 people with methamphetamine use disorder and 30 controls) completed a clinical interview, cognitive tasks, and provided a blood sample. *SLC18A1, SLC18A2, BDNF*, and *FAAH* SNPs were genotyped, and gene expression was assessed with real-time quantitative PCR.

**Results:**

*SLC18A1* Pro4Thr was associated with methamphetamine use disorder (OR = 6.22; *p* = .007). *SLC18A2* variants, rs363227 and rs363387, were negatively associated with methamphetamine use severity (*p* = .003) and positively associated with inhibitory control performance (*p* = .006), respectively. *BDNF* Val66Met was associated with the severity of use (*p* = .008). *SLC18A2* and *FAAH* mRNA levels were lower in people who use methamphetamine relative to controls (*p* = .021 and .010, respectively).

**Conclusions:**

*SLC18A1* is identified for the first time to play a potential role in methamphetamine use disorder. Lower levels of blood *SLC18A2* and *FAAH* mRNA in people with methamphetamine use disorder suggest reduced monoamine reuptake, recycling, or release, and higher anandamide levels in this clinical group, which may be potential therapeutic targets.

## Introduction

Methamphetamine is a widely used illicit drug globally (UNODC 2022). Chronic and repeated use can lead to substance use disorder (APA 2013). There are a wide range of risk factors associated with the development of substance use disorders, including genetics (Ducci and Goldman 2012; Whitesell et al. 2013). The heritability of stimulant use disorders is estimated to be ∼40% (Goldman et al. 2005). To date there have been three genome-wide association studies (GWASs) in people with methamphetamine use disorder (Uhl et al. 2008; Ikeda et al. 2013; Sun et al. 2021), but all were underpowered. Candidate-gene association studies offer a cost-effective alternative to GWASs, identifying specific sequence variations in genes of interest (e.g., single nucleotide polymorphisms (SNP) and deletions) to highlight potential molecular pathways and gene variants that may confer some susceptibility to the development of disorders. Notably, SNPs can influence promoter activity and expression of messenger RNA (mRNA) (Shastry 2009), which in turn can affect protein expression and localisation, and contribute to disease development. However a major limitation in the current candidate-gene association studies for methamphetamine use disorder is the inadequate rationale for gene and variant selection (Guerin et al. 2021b).

A thorough assessment of the literature identified four genes (*SLC18A1, SLC18A2, FAAH,* and *BDNF,*) potentially important in methamphetamine use (Supplementary Table S1). Meta-analytical evidence suggests that variants in the *FAAH* (rs324420) and *BDNF* (rs6265) genes may confer a risk for, and protection against, the disorder, respectively (Guerin et al. 2021b). In addition, vesicular monoamine transporter (VMAT) genes play a key role in regulating monoamine transport from cytosol to synaptic vesicles (Schuldiner et al. 1995). Two distinct transporters, VMAT1 and VMAT2, are encoded by *SLC18A1* and *SLC18A2*, respectively. While similar in structure, VMAT1 and VMAT2 have distinct properties with VMAT1 having a lower turnover number and lower affinity to monoamines relative to VMAT2. Historically, VMAT2 has received more attention in methamphetamine use, with a 10% reduction of VMAT2 binding availability reported in the striatal subregions of people who use methamphetamine (Johanson et al. 2006). In addition, several *SLC18A2* polymorphisms have been associated with substance use disorders (Schwab et al. 2005; Levran et al. 2015; Randesi et al. 2019). Likewise, *SLC18A1* polymorphisms are associated with several neuropsychiatric disorders, including alcohol use disorder (Bly 2005; Zhu et al. 2015; Vaht et al. 2016; Dutta et al. 2016). In people who use methamphetamine, *SLC18A1*/*SLC18A2* polymorphisms/mRNA levels and *BDNF* mRNA levels have never been assessed. One study investigated *FAAH* mRNA expression and reported lower levels of blood mRNA in people who use methamphetamine relative to controls (Zhang et al. 2020).

The primary aim of this study was to investigate SNP distribution and gene expression in four candidate genes between controls and people with methamphetamine use disorder. We hypothesised that some polymorphisms (i.e., *BDNF* Val66Met; *SLC18A1* Thr136Ile) would confer protection against methamphetamine use disorder, while other variants (i.e., *FAAH* Pro129Thr) would confer a risk. The secondary aim was to explore whether SNPs and gene expression were associated with (1) the severity of methamphetamine use; and (2) cognitive performance in people who use methamphetamine. This study focused on inhibitory control, given its treatment relevance as the most consistently impaired cognitive domain in people who use methamphetamine (Guerin et al. 2019; Potvin et al. 2018; Guerin et al. 2023; St. Peters et al. 2023).

## Methods

### Participants, study design and procedures

Fifty-nine people (29 people with methamphetamine use disorder and 30 controls) were recruited between March 2018 and December 2020 through referrals at the Department of Addiction Medicine at St Vincent’s Hospital Melbourne and from local community groups in Melbourne metropolitan area. All participants provided written informed consent before any assessment. The authors declare that all procedures contributing to this work comply with the ethical standards of the relevant national and institutional committees on human experimentation and with the Helsinki Declaration of 1975, as revised in 2008. All procedures were approved by St Vincent’s Hospital Melbourne Human Research Ethics Committee (HREC/17/SVHM/169).

Eligibility criteria are described elsewhere (Guerin et al. 2021a). In brief, people with methamphetamine use disorder were (1) aged between 18 and 50 years-old; and (2) met DSM-5 criteria for stimulant use disorder, methamphetamine-type, assessed with the MINI International Neuropsychiatric Interview for the DSM-5 (M.I.N.I 7.0.2.) (Sheehan et al. 1998). They were excluded if they (1) had a major neurological or medical illness; (2) a history of substance use disorder where methamphetamine was not the primary substance of abuse; or (3) if they were not able to provide informed consent. Controls were eligible to take part in the study if they (1) were aged between 18 and 50 years; and (2) had no history of substance use disorder. They were excluded if they had (1) a major neurological or medical illness; (2) a dependence on any substance excluding nicotine (other recreational use of substances were allowed); or (3) if they were not able to provide informed consent. The study was a cross-sectional two-group study conducted at the Department of Addiction Medicine at St Vincent’s Hospital Melbourne, Fitzroy, Australia. All participants were administered a clinical interview and cognitive task battery, followed by blood sample collection.

### Background measures

*A demographic questionnaire* was used to collect information on key demographic characteristics (i.e., age, sex, ethnicity).

*The Wechsler Test of Adult Reading* was used to assess premorbid verbal IQ (Wechsler, 1997). Participants read 50 irregularly spelled words. Each correct pronunciation was given a score of 1, with 50 as the maximum raw score. The raw score was then standardised by age (range 50–129).

*The Alcohol, Smoking and Substance Involvement Screening Test* (*ASSIST)* was used to assess severity, frequency, and high-risk substance use (Humeniuk et al. 2010). It assesses use of nine substances. A score of 4 or above reflects problematic use, and a score of 27 or above reflects high-risk use.

*The MINI International Neuropsychiatric Interview (M.I.N.I)* was used to confirm DSM-5 stimulant use disorder, methamphetamine-type diagnosis and assess for co-occurring psychiatric disorders (Sheehan et al. 1998).

### Cognitive measure

*The Word-Colour Stroop Task* was administered to measure inhibitory control and cognitive flexibility (Lezak et al. 2004). It consists of three trials. In the first trial (Baseline), participants were presented with coloured square and instructed to name the colour of the squares as fast as they can. In the second trial (Inhibition) participants were required to suppress a habitual response to read the words and instead name the ink colour of incongruently coloured words. In the third trial (Switching), participants were presented with two different rules such as reading the ink colour or the word and had to switch between the rules. For each index, the raw score was calculated as the time taken to complete the Inhibition or Switching trial minus the time taken to complete the Baseline. The performance index is the inverse score, calculated as 100/(raw performance score + 1), with final scores ranging from 0 to 100. Two participants in the methamphetamine group and one participant in the control group declined taking the test, or were unable to complete the task (e.g., due to colour-blindness).

### Blood collection

Blood (15 mL) was collected by venipuncture at the completion of the study using a Vacutainer Safety-Lok blood collection set (Becton, Dickinson and Company, NJ, USA) with a 22-gauge needle. Two samples were collected: whole blood in a 6 mL EDTA tube for genotype analysis; and whole blood in a 9 mL Tempus tube (Applied Biosystems, MA, USA) for gene expression analysis. The tubes were immediately stored at −80 °C following collection.

### Genotyping

DNA was extracted from whole blood using a DNEasy Blood & Tissue Kit (Qiagen, Netherland), according to manufacturer instructions (Carruthers et al. 2019). DNA concentration and purity were assessed using Nanodrop Spectrophotometers (ThermoFisher Scientific, MA, USA).

SNP genotyping was performed using the Agena MassArray system as per manufacturer’s standard protocols, and assays were designed using the Agena Assay Design Suite software (Agena San Diego, CA, USA (Gabriel et al. 2009)). Each SNP had forward and reverse PCR primers and iPLEX extension primers specific for the SNP of interest (Supplementary Table S2), and reactions were performed as per manufacturers protocols. Genotype analyses were performed on the Agena Typer Software Module (Agena San Diego), which automatically generates a report containing the SNP alleles in each sample. All genotype calls were made in real time during MALDI-TOF analysis. Allele frequency and adherence to Hardy-Weinberg equilibrium were assessed to ensure the validity of the results.

### Gene expression analysis (real-time quantitative PCR)

RNA was extracted using RNeasy mini kits (Qiagen) and reverse-transcribed to produce cDNA using Maxima H Minus first strand cDNA synthesis kit (Thermo Fisher Scientific) following manufacturer’s instructions. Real time quantitative PCR (qPCR) was used to measure the expression of specific genes as listed in Supplementary Table S3. The experiments were carried out in a QuantStudio 3 Real-time PCR system (Thermo Fisher Scientific), as described elsewhere (Truong et al. 2022). The Quant-iT OliGreen ssDNA Assay Kit was used to quantify the cDNA concentration in each sample as per the manufacturer’s instructions. Gene expression data was quantified using the ΔΔC_t_ method normalised to the measured cDNA concentration and housekeeping gene *HPRT* expression levels of each sample (Zbukvic et al. 2017; Bortolasci et al. 2020), and log2 transformed fold change normalised to the control group (Short et al. 2022).

### Statistical analyses

All statistical analyses were performed using SPSS Statistics 29 (IBM Corp., NY, USA). Independent samples t-tests and Chi-square analyses with Fisher’s exact test were used to compare methamphetamine use disorder and control groups for all demographics. Analysis of covariance (ANCOVA) using significantly different demographic characteristics as covariates was used to assess differences in cognitive measures between the two groups.

#### Genotype analyses

Chi-square analyses were used to calculate the significance for genotype distribution between controls and people with methamphetamine use disorder for each SNP. Odds ratios (OR) and 95% confidence intervals (CI) were calculated for alleles under all four inheritance models: co-dominant, dominant, recessive, and additive. Exploratory analyses of associations between genotypes, ASSIST score, and cognitive performance (inhibitory control and cognitive flexibility) were conducted in people with methamphetamine use disorder only. Datasets were not normally distributed (Kolmogorov–Smirnov test), hence non-parametric Mann-Whitney U tests were used. Bonferroni correction was employed (*p* < .017) to account for multiple comparisons.

#### Gene expression analyses

Datasets were not normally distributed (Kolmogorov–Smirnov test), hence non-parametric Mann-Whitney U tests were used to determine differences in fold change in mRNA expression normalised to control participants for *SLC18A2, FAAH,* and *BDNF*. For wildtype vs minor allele analyses, data from people with methamphetamine use disorder and controls were pooled, and Mann-Whitney U tests were used. Exploratory analyses of associations between mRNA levels, ASSIST score, and cognitive performance were examined using Pearson’s correlation analyses, only in people with methamphetamine use disorder with Bonferroni corrections for each measure (*p* < .017) to account for multiple comparisons. For correlation analyses, mRNA levels were assessed with a number of cycle threshold (CT) similar to previous studies (Bolotin et al. 2015; DE Francesco et al. 2019).

## Results

### Sample characteristics

Demographic characteristics, substance use characteristics, and Stroop performance of participants are in Table 1. There were no group differences in sex and ethnicity distribution, but people with methamphetamine use disorder were on average older than controls (*p* < .001) and had lower premorbid IQ (*p* = .003). Age and IQ were therefore used as covariates for the group comparisons for Stroop tasks. Controls performed better than people who use methamphetamine in the Stroop Inhibition (*p* = .010) and Switching (*p* = .003) trials. None of the control participants reported any lifetime use of amphetamines or other stimulants (Table 1).

**Table 1.** Demographic characteristics, substance use characteristics, and cognitive performance.

	Controls (*n* = 30)	Methamphetamine (*n* = 29)	Test; *p*-value
Age – years (±SD)	26.30 (±6.61)	32.34 (±6.75)	**t = −3.477; *p* < .001**
Females – no. (%)	19 (63.3)	12 (41.4)	*χ*^2^ = 2.850; *p* = .091
Premorbid IQ (±SD)	112.57 (±9.97)	104.41 (±10.44)	***t* = 3.068; *p* = .003**
Ethnicity – no. (%)			
Caucasian	20 (66.7)	21 (72.4)	χ2 = 3.008; *p* = .222
Asian	10 (33.3)	6 (20.7)	
Aboriginal/Torres Strait Islander	0 (0)	2 (6.9)	
ASSIST Amphetamine score (±SD)	0 (±0)	31.59 (±5.78)	–
Stroop (Inhibition) (±SD)	18.27 (±16.76)	10.82 (±4.86)	***F* = 7.214; *p* = .010**
Stroop (Switching) (±SD)	3.84 (±1.21)	2.56 (±1.42)	***F* = 9.862; *p* = .003**

Values in **bold** indicates a dependent variable with a significant group difference. For Stroop performance, higher score indicates a better performance and ANCOVA values with age and IQ as covariates is reported.

ASSISIT: Alcohol, Smoking, and Substance Involvement Screening Test; Methamphetamine: people with methamphetamine use disorder; SD: standard deviation.

### Genotype and allele distribution

The genotype distribution of *SLC18A1* rs2270641 (Pro4Thr) was significantly different between people with methamphetamine use disorder and controls (*χ*^2^ = 8.208; *p* = .017). There were no other significant differences (Table 2).

**Table 2.** Genotype frequencies and counts of variants of interest in people with methamphetamine use disorder and controls.

Gene	Marker	Control % (*n*)				Methamphetamine % (*n*)				*p*-value
		M/M	M/m	m/m	Total	M/M	M/m	m/m	Total	
*SLC18A1*	rs2270641	57.1 (16)	14.3 (4)	28.6 (8)	100 (28)	32.1 (9)	50.0 (14)	17.9 (5)	100 (28)	**.017**
	rs2270637	75.0 (21)	21.4 (6)	3.6 (1)	100 (28)	71.4 (20)	28.6 (8)	0.0 (0)	100 (28)	.519
	rs1390938	64.3 (18)	21.4 (6)	14.3 (4)	100 (28)	67.9 (19)	25.0 (7)	7.1 (2)	100 (28)	.680
*SLC18A2*	rs363227	72.4 (21)	17.2 (5)	10.3 (3)	100 (29)	60.7 (17)	35.7 (10)	3.6 (1)	100 (28)	.215
	rs363285	84.6 (11)	15.4 (2)	0.0 (0)	100 (13)	81.8 (9)	18.2 (2)	0.0 (0)	100 (11)	.855
	rs363333	82.1 (23)	14.3 (4)	3.6 (1)	100 (28)	75.0 (21)	21.4 (6)	3.6 (1)	100 (28)	.782
	rs363387	89.3 (25)	10.7 (3)	0.0 (0)	100 (28)	82.1 (23)	14.3 (4)	3.6 (1)	100 (28)	.542
	rs363276	73.1 (19)	19.2 (5)	7.7 (2)	100 (26)	53.6 (15)	39.3 (11)	7.1 (2)	100 (28)	.266
*BDNF*	rs6265	64.3 (18)	32.1 (9)	3.6 (1)	100 (28)	56.0 (14)	36.0 (9)	8.0 (2)	100 (25)	.717
*FAAH*	rs324420	51.7 (15)	44.8 (13)	3.4 (2)	100 (29)	64.3 (18)	32.1 (9)	3.6 (1)	100 (28)	.612

Values in **bold** are statistically significant (*p* < .05).

Supplementary Table S4 lists the allele frequencies, odds ratios (OR) and 95% confidence intervals (CI) in four inheritance models. For the co-dominant model, ORs were calculated by comparing distribution of heterozygous (M/m) and homozygous minor alleles (m/m) to the wildtype homozygous major allele genotype (M/M). *SLC18A1* rs2270641 (Pro4Thr) was significantly different between people who use methamphetamine and controls under the co-dominant model of inheritance, with the heterozygous variant (Pro/Thr) conferring greater risk of methamphetamine use disorder (OR = 6.22). There were no other significant differences.

### Allele distribution, methamphetamine use characteristics, and cognition

Table 3 shows the associations between genotypes and methamphetamine use severity (ASSIST score) and cognition (Stroop Inhibition and Stroop Switching) for each SNP of interest. There was a significant difference in ASSIST scores between *SLC18A2* rs363227 genotypes [*U* = 33.50, z = −2.840, *p* = .003], with people carrying the wildtype genotype having a higher score compared to people carrying at least one minor allele. There was also a significant difference in Stroop (Inhibition) scores between *SLC18A2* rs363387 genotypes [*U* = 93.00, *z* = 2.635, *p* = .003], with people carrying the wildtype genotype displaying worse performance compared to people carrying at least one minor allele. Lastly, there was a significant difference in ASSIST score between *BDNF* rs6265 (Val66Met) genotypes [*U* = 29.50, z = −2.617, *p* = .008], with people carrying the wildtype genotype having a higher score compared to people carrying at least one minor allele.

**Table 3.** Comparison between wildtype vs minor allele distribution and variables of interest.

		*n*	ASSIST Score		*n*	Stroop (Inhibition)		*n*	Stroop (Switching)	
			Mean (±SD)	Test; *p*-value		Mean (±SD)	Test; *p*-value		Mean (±SD)	Test; *p*-value
*SLC18A1* rs2270641 (Pro4Thr)	WT	9	33.67 (±3.81)	*U* = 58.00; *p* = .188	8	9.61 (±2.62)	*U* = 77.00; *p* = .807	7	2.49 (±0.79)	*U* = 48.00; *p* = .389
	Minor	19	30.42 (±6.43)		18	11.08 (±5.57)		18	2.51 (±1.55)	
*SLC18A1* rs2270637 (Ser98Thr)	WT	20	31.60 (±4.73)	*U* = 87.50; *p* = .709	19	10.89 (±5.48)	*U* = 68.00; *p* = .955	18	2.53 (±1.50)	*U* = 73.00; *p* = .574
	Minor	8	31.13 (±8.44)		7	9.90 (±2.66)		7	2.45 (±1.02)	
*SLC18A1* rs1390938 (Thr138Ile)	WT	19	30.74 (±6.44)	*U* = 104.00; *p* = .383	17	11.71 (±5.12)	*U* = 52.00; *p* = .200	17	2.63 (±1.59)	*U* = 65.00; *p* = .887
	Minor	9	33.00 (±4.30)		9	8.57 (±3.71)		8	2.25 (±0.69)	
*SLC18A2* rs363227	WT	17	33.94 (±6.27)	***U* = 33.50;** ***p* = .003**	15	11.10 (±3.54)	*U* = 121.00; *p* = .164	15	2.54 (±1.71)	*U* = 87.00; *p* = .531
	Minor	11	27.64 (±6.07)		11	9.98 (±6.36)		10	2.46 (±0.65)	
*SLC18A2 rs363285*	WT	9	31.78 (±5.17)	*U* = 10.50; *p* = .727	8	11.32 (±4.02)	*U* = 10.00; *p* = .711	8	3.56 (±1.82)	*U = 0.00; p = .044*
	Minor	2	33.00 (±7.07)		2	13.88 (±7.84)		2	2.08 (±0.24)	
*SLC18A2* *rs363333*	WT	21	31.57 (±6.19)	*U* = 66.50; *p* = .717	19	9.46 (±4.12)	*U* = 99.00; *p* = .063	18	2.35 (±0.83)	*U* = 63.00; *p* = 1.00
	Minor	7	31.14 (±5.11)		7	13.80 (±5.59)		7	2.90 (±2.29)	
*SLC18A2* *rs363387*	WT	23	31.39 (±6.35)	*U* = 54.50; *p* = .862	21	9.34 (±3.92)	***U* = 93.00;** ***p* = .006**	20	2.40 (±0.80)	*U* = 38.00; *p* = .447
	Minor	5	31.80 (±3.03)		5	16.03 (±4.99)		5	2.93 (±2.80)	
*SLC18A2* rs363276	WT	15	33.60 (±4.40)	*U = 54.00;p =* *.046*	13	10.77 (±3.14)	*U* = 155.00; *p* = .311	13	2.45 (±1.79)	*U* = 99.00; *p* = .270
	Minor	13	29.00 (±6.49)		13	10.47 (±6.25)		12	2.57 (±0.73)	
*BDNF* rs6265 (Val66Met)	WT	14	34.00 (±4.13)	***U* = 29.50;** ***p* = .008**	13	11.6 (±4.54)	*U* = 41.00; *p* = .148	13	2.49 (±1.77)	*U* = 75.00; *p* = .563
	Minor	11	27.73 (±6.54)		10	9.96 (± 5.63)		10	2.43 (±084)	
*FAAH* rs324420 (Pro129Thr)	WT	18	30.28 (±6.26)	*U* = 122.00; *p* = .133	16	11.12 (±5.87)	*U* = 75.00; *p* = .816	15	2.60 (±1.67)	*U* = 82.00; *p* = .723
	Minor	10	33.60 (±4.58)		10	9.84 (± 2.61)		10	2.36 (±0.74)	

### Gene expression

**Figure 1.**
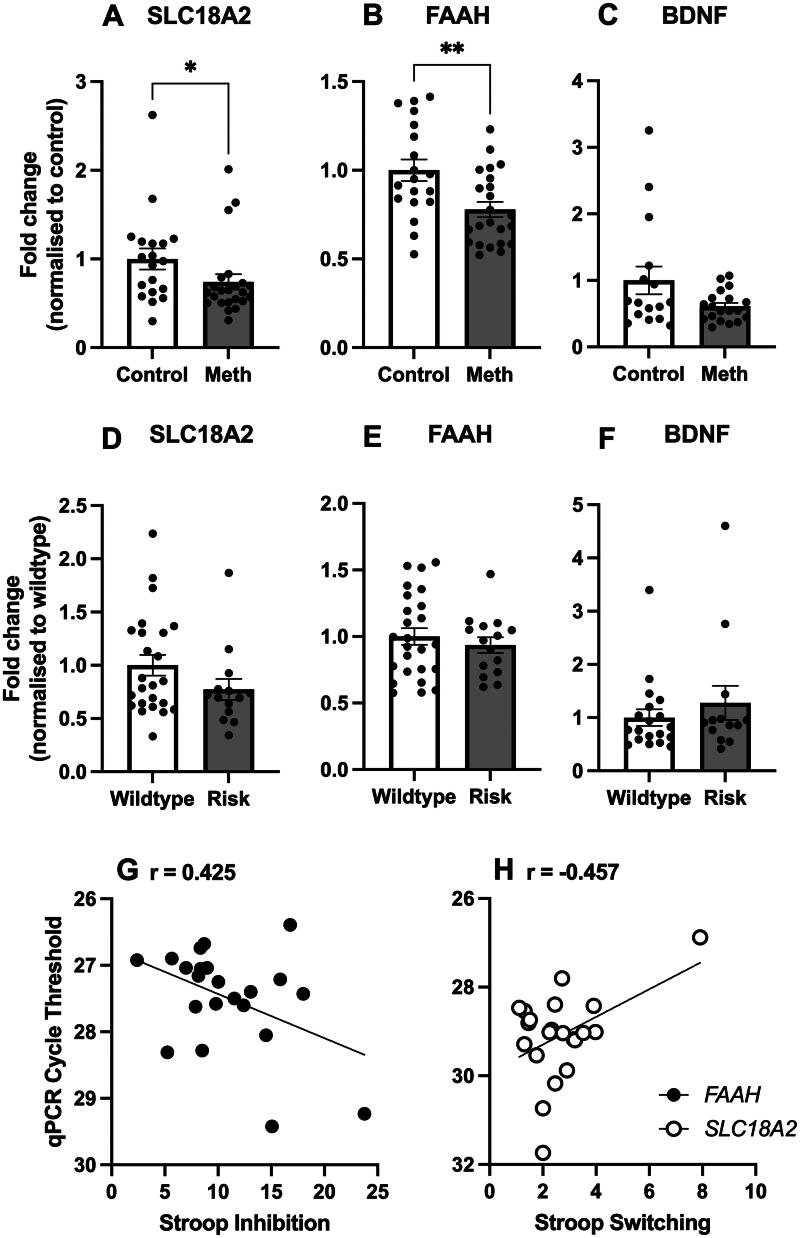
mRNA levels in people who use methamphetamine compared to controls. (A) *SLC18A2:* S*LC18A2* mRNA levels were significantly lower in people who use methamphetamine compared to controls (*p = .021). Control *n* = 19; Meth *n* = 23 (B) *FAAH: FAAH* mRNA levels were significantly lower in people who use methamphetamine compared to controls (**p = .010). Control *n* = 19; Meth *n* = 23 (C) *BDNF:* There were no differences in *BDNF* mRNA levels between groups. Control *n* = 16; Meth *n* = 19. mRNA levels as a function of genotype. (D) *SLC18A2:* There were no differences in *SLC18A2* mRNA levels between rs363276 genotypes. WT *n* = 24; Minor *n* = 14. (E) *FAAH:* There were no differences in *FAAH* mRNA levels between genotypes. WT *n* = 25; Minor *n* = 15. (F) *BDNF:* There was no significant difference in *BDNF* mRNA levels between genotypes. WT *n* = 19; Minor *n* = 13. Pearson’s correlation analyses between mRNA levels and cognition. (G) Stroop Inhibition. There was a positive association between performance in the Stroop Task (Inhibition) and *FAAH* mRNA qPCR cycle threshold (CT) (*p* = .048). (H) Stroop Switching. There was a negative association between performance in the Stroop Task (Switching) and *SLC18A2* mRNA qPCR CT (*p* = .037). Data represented as individual values and mean ± SEM when appropriate. CT: cycle threshold; Meth: people with methamphetamine use disorder; Minor: minor allele carriers computed as heterozygous (M/m) and homozygous minor alleles (m/m); SEM: standard error of the mean; WT: wildtype (M/M).

Log2 transformed fold change in mRNA expression normalised to control participants for *SLC18A2, FAAH,* and *BDNF* in peripheral blood samples is shown in Figure 1. *SLC18A1* mRNA could not be detected in peripheral blood samples. Compared to controls, *SLC18A2* levels [*U* = 127, z = -2.312, *p* = .021] (Figure 1A) and *FAAH* levels [*U* = 117, z = −2.565, *p* = .010] (Figure 1B) were significantly lower in people who use methamphetamine. There were no differences in *BNDF* levels between the groups [*U* = 117, z = −1.159, *p* = .257] (Figure 1C).

Differences in gene expression in peripheral blood samples between wildtype and minor allele carriers were also assessed. For *SLC18A2* expression, the rs363276 SNP in particular was selected given its role in other substance dependence (Levran et al. 2015; Randesi et al. 2019), and effects on mRNA abundance in the brain (Bharadwaj et al. 2018). There were no differences in gene expression between genotypes for *SLC18A2* [*U* = 121, z = −1.422, *p* = .161] (Figure 1D), *FAAH* [*U* = 171, z = −0.461, *p* = .659] (Figure 1E), or *BDNF* [*U* = 108, z = −0.595, *p* = .570].

### Gene expression, methamphetamine use characteristics, and cognition

Similar to previous studies (Bolotin et al. 2015; De Francesco et al. 2019), correlations between mRNA levels - assessed with raw cycle threshold (CT) value - and methamphetamine use characteristics (ASSIST score) and cognition (Stroop Inhibition and Stroop Switching) were explored for people with methamphetamine use disorder (Table 4).

**Table 4.** Pearson’s correlation analyses between mRNA levels, methamphetamine use characteristics, and cognitive performance people with methamphetamine use disorder.

	*SLC18A2* mRNA (CT)		*FAAH* mRNA (CT)		*BDNF* mRNA (CT)	
	*r*	*p*	*r*	*p*	*r*	*p*
ASSIST Score	−0.059	.790	0.073	.739	−0.250	.302
Stroop (Inhibition)	0.127	.572	*0.425*	*.048*	0.222	.375
Stroop (Switching)	*−0.457*	*.037*	−0.322	.154	−0.215	.391

Values in bold are significant at *p* = .017 (Bonferroni corrected). Values *in italic* are significant at raw *p* < .05. CT: qPCR cycle threshold.

*FAAH* CT values were positively associated with Stroop Inhibition performance (*r* = 0.425, *p* = .048), indicating that lower levels of peripheral *FAAH* mRNA were associated with better inhibitory control (Figure 1G). *SLC18A2* CT values were negatively associated with Stroop Switching performance (*r* = −0.457, *p* = .037), indicating that higher levels of peripheral *SLC18A2* mRNA were associated with better cognitive flexibility (Figure 1H). Notably, none of the significant associations survived Bonferroni corrections.

## Discussion

In this investigation of candidate gene SNPs and expression, the *SLC18A1* Pro4Thr variant was positively associated with methamphetamine use disorder. Two *SLC18A2* variants, rs363227 and rs363387, were associated with the severity of methamphetamine use and inhibitory control performance, respectively. Contrary to our hypothesis, *BDNF* Val66Met and *FAAH* Pro129Thr were not associated with methamphetamine use disorder in this sample. However, *BDNF* Val66Met was associated with the severity of use. *SLC18A2* blood mRNA levels were lower in people who use methamphetamine relative to controls. In people who use methamphetamine, lower *FAAH* mRNA expression (i.e., higher CT value) was associated with better inhibitory control performance while higher *SLC18A2* mRNA expression (i.e., lower CT value) was associated with better cognitive flexibility.

### SLC18A1

This study provides evidence of people who use methamphetamine to be ∼6 times more likely to carry the *SLC18A1* rs2270641 heterozygous genotype (Pro/Thr) relative to controls. *SLC18A1* codes for VMAT1, a protein involved in monoamine reuptake and release (Schuldiner et al. 1995) with functional outcomes in the dopaminergic system (Tunbridge et al. 2019). rs2270641 results in the substitution of a threonine (Thr) for a proline (Pro), and is associated with schizophrenia in Caucasian people (Bly 2005). While it is speculated that rs2270641 may affect uptake and/or release of neurotransmitter from vesicles (Bly 2005), its precise biological function is unknown. There were no associations between any of the other *SLC18A1* variants and severity of methamphetamine use or performance in the cognitive tasks. Lastly, while *SLC18A1* expression has previously been detected in other peripheral tissues including the lungs (Lehrer and Rheinstein 2018), it could not be detected in blood in the present study.

### SLC18A2

There were no group differences for any of the *SLC18A2* variants. However, *SLC18A2* rs363227 was associated with the severity of methamphetamine use, with the wild-type genotype (C/C) associated with higher ASSIST scores. While the precise biological function of rs363227 is unknown, it is possible that the wild-type genotype reduces VMAT2 function compared to the minor allele variant, thus blocking the reuptake of cytosolic dopamine and leading to reduced release of dopamine in the synaptic cleft upon methamphetamine exposure (Stahl 2018). This notion is consistent with other published reports indicating a loss of function of SLC transport proteins as a result of disease-associated mutations (Hamilton et al. 2013). People with rs363227 wild-type may therefore need to use a higher dose to achieve the rewarding effect of methamphetamine, which may lead to increased methamphetamine use. Conversely, a previous report found an association between the minor allele (T-allele) of rs363227 and psychosis liability (Simons and Van Winkel 2013), suggesting that while the minor allele confers protection against methamphetamine use severity, it may increase the risk for psychosis. There was also an association between rs363387 wild-type (T/T) and lower performance on the Stroop task, indicating poorer inhibitory control. The rs363387 wild-type is associated with a risk of alcohol dependence (Schwab et al. 2005; Fehr et al. 2013), another substance use disorder often characterised by cognitive impairments (Perry 2016). Reduced levels of the neurotransmitter dopamine can contribute to reductions of inhibitory control caused by other drugs of dependence (Luijten et al. 2013), therefore, it is possible that the wild-type rs363387 results in reduced VMAT2 function, resulting in decreased levels of dopamine. Developing or repurposing pharmacotherapies targeting monoamine reuptake by enhancing VMAT2 function could therefore be a promising new intervention to reduce methamphetamine use in people carrying the rs363227 or rs363227 mutations. For example, preclinical studies suggest lithium increases neural VMAT2 (Cordeiro et al. 2002) and reverse the neural and behavioural effects of methamphetamine (Ago et al. 2012; Wu et al. 2015).

A group difference in blood *SLC18A2* mRNA levels was observed for the first time, with people in the methamphetamine group having lower levels than controls. This is consistent with a positron emission tomography study which found a 10% reduction of VMAT2 binding availability in the brain of people who use methamphetamine (Johanson et al. 2006). Notably, there were no differences in blood mRNA levels between rs363276 genotypes, which contrasts with a study which found a genotype effect of rs363276 on mRNA abundance in the prefrontal cortex and amygdala of post-mortem brains with the C-allele resulting in higher levels (Bharadwaj et al. 2018). Genotype effects on mRNA abundance may be tissue specific. Interestingly, higher *SLC18A2* mRNA expression in the blood was associated with better cognitive flexibility, which is consistent with a study that reported a positive association between levels of *SLC18A2* and performance on a cognitive task in people with Body Mass Index ≤35 kg/m^2^ (Oliveras-Cañellas et al. 2023). In the present study, this correlation did not survive correction for multiple comparisons.

### BDNF

Contrary to our hypothesis, *BDNF* rs6265 did not distinguish people with methamphetamine use disorder vs controls in the present study. A previous meta-analysis comprising a total of 4,674 participants reported a protective effect of rs6265 (Guerin et al. 2021b). Our sample size was limited to 59, and thus may explain the discrepant findings. Nonetheless, there was a significant association between *rs6265* and the severity of methamphetamine use. Specifically, participants carrying the homozygous major alleles (Val/Val) reported higher ASSIST scores compared to carriers of the minor-allele (Met), suggesting that protection conferred by *rs6265* may be due to lower severity of use in people carrying at least one minor allele. Lower levels of the BDNF protein caused by the Met variant may reduce the rewarding effect of methamphetamine (Egan et al. 2003; Ghitza et al. 2010), and in turn reduce the severity and frequency of use. It is unlikely that the Met allele exerts its protective effect by reducing methamphetamine-associated anxiety during withdrawal because the Met allele is associated with anxiety disorders and anxiety-like behaviours (Moreira et al. 2015; Chen et al. 2017; González-Castro et al. 2019; Tomasi et al. 2021).

In contrast to previous reports (Su et al. 2014, 2015), we did not find an association between rs6265 and cognition, which may be due to the differences in demographic characteristics of the studies. Previous studies predominantly assessed males with only 16.5%–21% female participants compared to 41.4% in the present study. There is evidence for sex-specific genetic effects on human disease (Ober et al. 2008). In addition, a sexually-dimorphic effect of BDNF deficiency on impulse behaviours in mice exposed to methamphetamine in young adulthood has been observed, with *BDNF* genotypes having no effects on prepulse inhibition in females (Manning and Van Den Buuse 2013).

While no studies have investigated blood *BDNF* mRNA levels in people who use methamphetamine, higher serum BDNF protein levels have been reported in people with methamphetamine dependence (Su et al. 2015; Ren et al. 2016; Ren et al. 2017). Notably, methamphetamine withdrawal and abstinence were associated with reductions in BDNF protein levels (Chen et al. 2014; Ren et al. 2016). Given that gene expression is one of many processes involved in protein production, future studies should aim to assess the relationship between *BDNF* gene and protein expression in people with methamphetamine use disorder, and their association with cognitive and clinical correlates.

### FAAH

FAAH is an enzyme which breaks down the endocannabinoid anandamide (Deutsch and Chin 1993). The rs324420 variant is a missense mutation converting proline (Pro/C-allele) to threonine (Thr/A-allele), which reduces FAAH’s catalytic activity and reduces its stability, resulting in increased brain anandamide levels (Chiang et al. 2004; Dincheva et al. 2015). While a strong association between *FAAH* rs324420 and methamphetamine use disorder has been reported (Guerin et al. 2021b), this was not replicated by the present study, possibly due to the sample size. However, blood *FAAH* mRNA levels were significantly lower in the methamphetamine group relative to controls in this study, consistent with a previous report (Zhang et al. 2020). Further, there were no differences in mRNA expression between genotypes in neither Zhang et al. (2020) nor the present study.

*FAAH* mRNA levels were associated with performance on the Stroop task (inhibition trial) in people who use methamphetamine, with lower levels resulting in better performance. While we did not assess protein expression, it is possible that lower *FAAH* mRNA expression results in reduced levels of FAAH protein, and therefore increased levels of anandamide. Rodent research suggests that inhibition of FAAH may lead to cognitive improvement (Hlavacova et al. 2015) and prevent memory impairment (Hasanein and Teimuri Far 2015). In addition, acute anandamide administration prevents impairments in recognition and non-associative emotional memory (Moreira-Silva et al. 2018), and lowers impulsivity in rats (Panlilio et al. 2009). Elevated levels of anandamide may be protective against cognitive impairments often observed in people who use methamphetamine (Potvin et al. 2018; Guerin et al. 2019). FAAH inhibitors such as LEI-401 (Mock et al. 2020) could be a promising pharmacological approach to target some of the most common cognitive deficits observed in people who use methamphetamine.

### Limitations

Firstly, the small sample size in the present study was lower than expected in a candidate-gene association study. Meaningful associations possibly went undetected, and the genotype results from this study should be taken with great caution and seen as preliminary to inform future larger hypothesis-driven studies. Future studies should also aim to assess peripheral protein expression to obtain a better understanding of the role played by these genes of interest in methamphetamine use disorder development. It should also be noted that none of the SNPs investigated were top hits on the three GWASs conducted in people who use methamphetamine to date, although there were significant limitations to these GWASs (Uhl et al. 2008; Ikeda et al. 2013; Sun et al. 2021). It could indicate that the present target genes have a weaker influence on phenotype than other significant loci, or that these genes interact with other environmental factors to result in disease development (Sato et al. 2022). *SLC18A1* mRNA could not be detected in the blood. Given it is expressed in the human brain (Peter et al. 1995; Erickson et al. 1996), post-mortem analyses would be more informative in future studies. Lastly, we made our best effort to account for key covariates (e.g., age, IQ). We do, however, acknowledge the potential of bias in some of the results obtained due to the residual confounding with the sample size limiting the control of every possible factor.

## Conclusions

A potential role of the *SLC18A1* Pro4Thr variant in conferring a risk for methamphetamine use disorder was observed in this study. Consistent with the literature, *BDNF* Val66Met was associated with lower severity of methamphetamine use in this sample. *SLC18A2* variants have also been identified to be associated with severity of methamphetamine use and inhibitory control. Both *SLC18A2* and *FAAH* blood mRNA levels were lower in people who use methamphetamine relative to controls, with higher *SLC18A2* levels associated with better cognitive flexibility, whereas lower *FAAH* expression was associated with better inhibitory control in people who use methamphetamine. Despite some limitations, these preliminary results can inform larger hypothesis-driven clinical and preclinical studies to further characterise the contribution of these genes to the development of methamphetamine use disorder. In addition, future investigation of the function of the novel polymorphisms identified in this study will allow for the development of pharmacological treatments directly targeting the function of these mutations, thus providing new promising treatments for methamphetamine use disorder.

## Supplementary Material

GuerinEtAl_Supplementary_Material_Revision (final).docx
