# Supplementary material for: Associations between methamphetamine use disorder and SLC18A1, SLC18A2, BDNF, and FAAH gene sequence variants and expression levels
Source: Dialogues Clin Neurosci. 2024 Oct 12;26(1):64–76. doi: 10.1080/19585969.2024.2413476 (PMC11486062; doi:10.1080/19585969.2024.2413476)
Supplement: GuerinEtAl_Supplementary_Material_Revision (final).docx [file TDCN_A_2413476_SM2841.docx]

**Supplementary Table S1 – Candidate genes investigated and rationale.** M = major allele; m = minor allele

| **Gene Name** | **Marker** | **Alleles (M>m)** | **Location** | **Gene function** | **Rationale** |
| --- | --- | --- | --- | --- | --- |
| ***SLC18A1***  Solute carrier family 18 member 1 | rs2270641 | T>G  Thr>Pro | 8p21.3 | Regulates monoamine transport from cytosol to synaptic vesicles | Associated with schizophrenia (24) |
|  | rs2270637 | C>G  Ser>Thr |  |  | Associated with schizophrenia (25) |
|  | rs1390938 | G>A  Thr>Ile |  |  | Functional variant - greater binding and neurotransmitter uptake (23)  Associated with alcohol use disorder (26-28) |
| ***SLC18A2***  Solute carrier family 18 member 2 | rs363227 | C>T | 10q25.3 | Regulates monoamine transport from cytosol to synaptic vesicles | Associated with schizophrenia (19) |
|  | rs363285 | A>C |  |  | Associated with poor executive function in schizophrenia (36) |
|  | rs363333 | T>C |  |  | Associated with alcohol dependence (18) |
|  | rs363387 | T>G  Thr>Thr |  |  | Associated with alcohol dependence (18, 20) |
|  | rs363276 | C>T |  |  | Reduced mRNA abundance in prefrontal cortex and amygdala (37)  Associated with cocaine dependence (21) |
| ***BDNF***  Brain-derived neurotrophic factor | rs6265 | G>A  Val>Met | 11p14.1 | Growth and differentiation of new neurons and synapses | Associated with methamphetamine use disorder [OR = 0.78 95%CI (0.69-0.88)] (10) |
| ***FAAH***  Fatty acid amide hydrolase | rs324420 | C>A  Pro>Thr | 1p33 | Degrades anandamide | Associated with methamphetamine use disorder [OR = 1.64 95%CI (1.40-1.92)] (10) |

**Supplementary Table S2.** Primers for single nucleotide polymorphisms of interest

| **Gene** | **Marker** | **Forward Primer (5’→3’)** | **Reverse Primer (3’→5’)** | **Extend Primer (5’→3’)** |
| --- | --- | --- | --- | --- |
| ***SLC18A1*** | rs2270641 | ACGTTGGATGTCCTTCAGCAACCGCTGGG | ACGTTGGATGACAGGACCTTCCCTAAGAGC | CGGGGAGCATCCAGAATGG |
|  | rs2270637 | ACGTTGGATGTTCAACAACAACACCGTGGC | ACGTTGGATGTGCTGGCAGTGTCATTCATC | TTGAAGAAAGCGTACCTA |
|  | rs1390938 | ACGTTGGATGAACAACTGCTTGCAAGGCAC | ACGTTGGATGTTGAAGCAAACAGAACCCCG | GTTTCTTGGAGGAAGAGA |
| ***SLC18A2*** | rs363227 | ACGTTGGATGTGCTACAGAAATCCAGACGG | ACGTTGGATGAAGATGCTCTGGAAGCTGTC | TACAGGCCAGTGCACAC |
|  | rs363285 | ACGTTGGATGTTGACAACCATCAGGACAAC | ACGTTGGATGTGAGATATGTAACTAGCCAC | GATGGAAGGATAACTTCCTGG |
|  | rs363333 | ACGTTGGATGATGCACTTACGAAACCTGGC | ACGTTGGATGTAAAGAGGACCTCTTCCACC | GGGAAACCTGGCATTTAGACA |
|  | rs363387 | ACGTTGGATGTGGTGATTCTTGCCCAAAGC | ACGTTGGATGGTTGGAGTCAGCATTTTATG | AGATTGTTCCCACTTGTC |
|  | rs363276 | ACGTTGGATGTGCATACTTAACTGACCAGC | ACGTTGGATGAAACTTCACCTGAAACAGGC | TCCAAGCATTTGTCAATTCACC |
| ***BDNF*** | rs6265 | ACGTTGGATGCTTCATTGGGCCGAACTTTC | ACGTTGGATGGCTTGACATCATTGGCTGAC | GTTCCTCATCCAACAGCTCTTCTATCA |
| ***FAAH*** | rs324420 | ACGTTGGATGTATCTGGCTGACTGTGAGAC | ACGTTGGATGACCTTGTAGGTGAAGCACTC | GAGCAGGCCCTGCCTTG |

**Supplementary Table S3.** Primers for genes of interest and housekeeping genes

| **Gene** | **Forward Primer (5’-3’)** | **Reverse Primer (3’-5’)** |
| --- | --- | --- |
| ***SLC18A1*** | AGCTGGCAAGTTGAAAAGCG | ATG GAA GAG GGG AGG GAG TC |
| ***SLC18A2*** | ACAGAAATCCAGACGGCCAG | GGGACAGTCGGAAGGAACAG |
| ***BDNF*** | TTTGGTTGCATGAAGGCTGC | GCCGAACTTTCTGGTCCTCA |
| ***FAAH*** | AAGGTGATTTCGTGGACCCC | CGGTACACCTCGATCTCGTG |
| ***HPRT*** | CCTGGCGTCGTGATTAGTGA | AGGGCTACAATGTGATGGCC |

**Supplementary Table S4.** **Allele counts of variants of interest in people with methamphetamine use disorder and controls in four inheritance models.** Values in **bold** are statistically significant (p < 0.05).

| **Gene/Marker** | **Model** | **Allele** | **Meth** | **Control** | **OR** | **95% CI** | **p-value** |
| --- | --- | --- | --- | --- | --- | --- | --- |
| ***SLC18A1***  **rs2270641**  **(Pro4Thr)** | Co-dominant | M/M | 9 | 16 | 1.00 (ref) |  |  |
|  |  | M/m | 14 | 4 | 6.22 | 1.57-24.71 | **0.007** |
|  |  | m/m | 5 | 8 | 1.11 | 0.28-4.43 | 0.881 |
|  | Additive | M | 32 | 36 | 1.00 (ref) |  |  |
|  |  | m | 24 | 20 | 1.35 | 0.63-2.89 | 0.439 |
|  | Dominant | M/M | 9 | 16 | 1.00 (ref) |  |  |
|  |  | M/m+m/m | 19 | 12 | 2.82 | 0.95-8.38 | 0.060 |
|  | Recessive | M/M+M/m | 23 | 20 | 1.00 (ref) |  |  |
|  |  | m/m | 5 | 8 | 0.54 | 0.15-1.93 | 0.342 |
| ***SLC18A1***  **rs2270637**  **(Ser98Thr)** | Co-dominant | M/M | 20 | 21 | 1.00 (ref) |  |  |
|  |  | M/m | 8 | 6 | 1.40 | 0.41-4.76 | 0.589 |
|  |  | m/m | 0 | 1 | - | - | 0.335 |
|  | Additive | M | 48 | 48 | 1.00 (ref) |  |  |
|  |  | m | 8 | 8 | 1.00 | 0.35-2.88 | 1.00 |
|  | Dominant | M/M | 20 | 21 | 1.00 (ref) |  |  |
|  |  | M/m+m/m | 8 | 7 | 1.20 | 0.37-3.92 | 0.763 |
|  | Recessive | M/M+M/m | 28 | 27 | 1.00 (ref) |  |  |
|  |  | m/m | 0 | 1 | 0.49 | 0.38-0.64 | 0.313 |
| ***SLC18A1***  **rs1390938**  **(Thr138Ile)** | Co-dominant | M/M | 19 | 18 | 1.00 (ref) |  |  |
|  |  | M/m | 7 | 6 | 1.11 | 0.31-3.92 | 0.877 |
|  |  | m/m | 2 | 4 | 0.47 | 0.08-2.91 | 0.413 |
|  | Additive | M | 45 | 42 | 1.00 (ref) |  |  |
|  |  | m | 11 | 14 | 0.73 | 0.30-1.79 | 0.496 |
|  | Dominant | M/M | 19 | 18 | 1.00 (ref) |  |  |
|  |  | M/m+m/m | 9 | 10 | 0.85 | 0.28-2.58 | 0.778 |
|  | Recessive | M/M+M/m | 26 | 24 | 1.00 (ref) |  |  |
|  |  | m/m | 2 | 4 | 0.46 | 0.08-2.75 | 0.388 |
|  |  | m/m | 1 | 2 | 0.46 | 0.04-5.43 | 0.531 |
| ***SLC18A2***  **rs363227** | Co-dominant | M/M | 17 | 21 | 1.00 (ref) |  |  |
|  |  | M/m | 10 | 5 | 2.47 | 0.71-8.62 | 0.150 |
|  |  | m/m | 1 | 3 | 0.41 | 0.04-4.33 | 0.448 |
|  | Additive | M | 44 | 47 | 1.00 (ref) |  |  |
|  |  | m | 12 | 11 | 1.17 | 0.47-2.91 | 0.743 |
|  | Dominant | M/M | 17 | 21 | 1.00 (ref) |  |  |
|  |  | M/m+m/m | 11 | 8 | 0.32 | 0.03-3.29 | 0.349 |
|  | Recessive | M/M+M/m | 27 | 26 | 1.00 (ref) |  |  |
|  |  | m/m | 1 | 3 | 0.54 | 0.15-1.93 | 0.317 |
|  |  | m/m | 11 | 11 | 1.00 | 0.34-2.92 | 1.000 |
| ***SLC18A2* rs363285** | Co-dominant | M/M | 9 | 11 | 1.00 (ref) |  |  |
|  |  | M/m | 2 | 2 | 1.22 | 0.14-10.48 | 0.855 |
|  |  | m/m | 0 | 0 | - | - | - |
|  | Additive | M | 20 | 24 | 1.00 (ref) |  |  |
|  |  | m | 2 | 2 | 1.20 | 0.16-9.30 | 0.861 |
|  | Dominant | M/M | 9 | 11 | 1.00 (ref) |  |  |
|  |  | M/m+m/m | 2 | 2 | 1.22 | 0.14-10.48 | 0.855 |
|  | Recessive | M/M+M/m | 11 | 13 | 1.00 (ref) |  |  |
|  |  | m/m | 0 | 0 | - | - | - |
| ***SLC18A2***  **rs363333** | Co-dominant | M/M | 21 | 23 | 1.00 (ref) |  |  |
|  |  | M/m | 6 | 4 | 1.64 | 0.41-6.64 | 0.484 |
|  |  | m/m | 1 | 1 | 1.10 | 0.06-18.64 | 0.950 |
|  | Additive | M | 48 | 50 | 1.00 (ref) |  |  |
|  |  | m | 8 | 6 | 1.39 | 0.45-4.30 | 0.568 |
|  | Dominant | M/M | 21 | 23 | 1.00 (ref) |  |  |
|  |  | M/m+m/m | 7 | 5 | 1.53 | 0.42-5.57 | 0.515 |
|  | Recessive | M/M+M/m | 27 | 27 | 1.00 (ref) |  |  |
|  |  | m/m | 1 | 1 | 1.00 | 0.06-16.82 | 1.00 |
| ***SLC18A2***  **rs363387** | Co-dominant | M/M | 23 | 25 | 1.00 (ref) |  |  |
|  |  | M/m | 4 | 3 | 1.45 | 0.29-7.18 | 0.648 |
|  |  | m/m | 1 | 0 | - | - | 0.302 |
|  | Additive | M | 50 | 53 | 1.00 (ref) |  |  |
|  |  | m | 6 | 3 | 2.12 | 0.50-8.94 | 0.297 |
|  | Dominant | M/M | 23 | 25 | 1.00 (ref) |  |  |
|  |  | M/m+m/m | 5 | 3 | 1.81 | 0.39-8.44 | 0.445 |
|  | Recessive | M/M+M/m | 27 | 28 | 1.00 (ref) |  |  |
|  |  | m/m | 1 | 0 | - | - | 0.313 |
| ***SLC18A2***  **rs363276** | Co-dominant | M/M | 15 | 19 | 1.00 (ref) |  |  |
|  |  | M/m | 11 | 5 | 2.79 | 0.79-9.78 | 0.104 |
|  |  | m/m | 2 | 2 | 1.27 | 0.16-10.08 | 0.823 |
|  | Additive | M | 41 | 43 | 1.00 (ref) |  |  |
|  |  | m | 15 | 9 | 1.75 | 0.69-4.43 | 0.236 |
|  | Dominant | M/M | 15 | 19 | 1.00 (ref) |  |  |
|  |  | M/m+m/m | 13 | 7 | 2.35 | 0.75-7.36 | 0.138 |
|  | Recessive | M/M+M/m | 26 | 24 | 1.00 (ref) |  |  |
|  |  | m/m | 2 | 2 | 0.92 | 0.12-7.08 | 0.939 |
| ***BDNF* rs6265**  **(Val66Met)** | Co-dominant | M/M | 14 | 18 | 1.00 (ref) |  |  |
|  |  | M/m | 9 | 9 | 1.29 | 0.40-4.09 | 0.670 |
|  |  | m/m | 2 | 1 | 2.57 | 0.21-31.33 | 0.446 |
|  | Additive | M | 37 | 45 | 1.00 (ref) |  |  |
|  |  | m | 13 | 11 | 1.44 | 0.58-3.58 | 0.435 |
|  | Dominant | M/M | 14 | 18 | 1.00 (ref) |  |  |
|  |  | M/m+m/m | 11 | 10 | 1.41 | 0.47-4.27 | 0.538 |
|  | Recessive | M/M+M/m | 23 | 27 | 1.00 (ref) |  |  |
|  |  | m/m | 2 | 1 | 2.35 | 0.20-27.59 | 0.486 |
| ***FAAH* rs324420**  **(Pro129Thr)** | Co-dominant | M/M | 18 | 15 | 1.00 (ref) |  |  |
|  |  | M/m | 9 | 13 | 0.58 | 0.19-1.72 | 0.322 |
|  |  | m/m | 1 | 1 | 0.83 | 0.05-14.48 | 0.900 |
|  | Additive | M | 45 | 43 | 1.00 (ref) |  |  |
|  |  | m | 11 | 15 | 0.70 | 0.29-1.70 | 0.439 |
|  | Dominant | M/M | 18 | 15 | 1.00 (ref) |  |  |
|  |  | M/m+m/m | 10 | 14 | 0.60 | 0.21-1.72 | 0.337 |
|  | Recessive | M/M+M/m | 27 | 28 | 1.00 (ref) |  |  |
|  |  | m/m | 1 | 1 | 1.04 | 0.06-17.43 | 0.980 |

CI: confidence interval; M: major allele; m: minor allele; Meth; methamphetamine; OR: odds ratio.
